# Supplementary material for: Comparison of Patient Survival According to Erythropoiesis-Stimulating Agent Type of Treatment in Maintenance Hemodialysis Patients
Source: J Clin Med. 2023 Jan 12;12(2):625. doi: 10.3390/jcm12020625 (PMC9861937; doi:10.3390/jcm12020625)
Supplement: Supplementary file 1 [file jcm-12-00625-s001.zip › jcm-2133631-supplementary.pdf]

Supplementary Materials

Table S1. Cox regression analyses for patient survival in HD patients < 65 years old.
Table S2. Cox regression analyses for patient survival in HD patients ≥ 65 years.
Table S3. Cox regression analyses for patient survival in male HD patients.
Table S4. Cox regression analyses for patient survival in female HD patients.
Table S5. Cox regression analyses for patient survival according to ESA dose tertile in EP group.
Table S6. Cox regression analyses for patient survival according to ESA dose tertile in DP group.
Table S7. Cox regression analyses for patient survival according to ESA dose tertile in MR group.
Table S8. Linear regression analysis to identify factors associated with ERI.

Table S1. Cox regression analyses for patient survival in HD patients <65 years old.

	Univariate		Multivariate	
	**HR (95% CI)**	***p***	**HR (95% CI)**	***p***
Ref: EP group				
DP group	0.98 (0.92–1.04)	0.494	1.01 (0.94–1.08)	0.868
MR group	0.93 (0.82–1.05)	0.215	0.92 (0.79–1.06)	0.224
Ref: DP group				
MR group	0.95 (0.83–1.08)	0.403	0.91 (0.78–1.06)	0.224
Age (per 1 year increase)	1.07 (1.06–1.07)	<0.001	1.05 (1.04–1.05)	<0.001
Sex (ref: male)	0.79 (0.75–0.83)	<0.001	0.67 (0.62–0.73)	<0.001
CCI score (per 1 score increase)	1.17 (1.16–1.18)	<0.001	1.10 (1.08–1.11)	<0.001
Body mass index (per 1 kg/m^2 increase)	0.99 (0.98–0.99)	0.001	0.99 (0.98–0.99)	0.029
Underlying etiology of ESRD (ref: DM)				
Glomerulonephritis	0.27 (0.24–0.30)	<0.001	0.39 (0.34–0.45)	<0.001
Hypertension	0.49 (0.46–0.52)	<0.001	0.58 (0.53–0.63)	<0.001
Others	0.44 (0.40–0.49)	<0.001	0.61 (0.54–0.69)	<0.001
Unknown	0.44 (0.40–0.48)	<0.001	0.58 (0.52–0.66)	<0.001
Hemoglobin (per 1 g/dL increase)	0.79 (0.77–0.82)	<0.001	0.89 (0.85–0.93)	<0.001
Serum creatinine (per 1 mg/dL increase)	0.89 (0.89–0.90)	<0.001	0.93 (0.92–0.95)	<0.001
SBP (per 1 mmHg increase)	1.01 (1.01–1.01)	<0.001	1.00 (1.00–1.01)	<0.001
DBP (per 1 mmHg increase)	0.99 (0.99–0.99)	0.001	1.00 (1.00–1.01)	0.504
Serum calcium (per 1 mg/dL increase)	0.99 (0.96–1.01)	0.344	1.09 (1.05–1.13)	<0.001
Serum phosphorus (per 1 mg/dL increase)	0.94 (0.92–0.95)	<0.001	1.08 (1.05–1.10)	<0.001
Kt/V_{urea} (per 1 unit increase)	0.67 (0.61–0.74)	<0.001	0.70 (0.60–0.80)	<0.001
Serum albumin (per 1 g/dL increase)	0.44 (0.41–0.47)	<0.001	0.61 (0.56–0.67)	<0.001
HD vintage (per 1 day increase)	1.00 (1.00–1.00)	<0.001	1.00 (1.00–1.00)	<0.001
Ultrafiltration volume (per 1 L increase)	1.05 (1.03–1.08)	<0.001	1.04 (1.01–1.08)	0.005
ERI (per 1 unit increase)	1.02 (1.01–1.02)	<0.001	1.04 (1.03–1.06)	<0.001
ESA dose (per 1 unit/week increase)	1.00 (1.00–1.00)	<0.001	1.00 (1.00–1.00)	<0.001

Multivariate analysis was adjusted for group, age, sex, CCI score, body mass index, underlying etiology of ESRD, hemoglobin, serum creatinine, SBP, DBP, serum calcium, serum phosphorus, Kt/V_{urea}, serum albumin, HD vintage, ultrafiltration volume, ERI, and ESA dose, and was performed using enter mode. **Abbreviations**: HD, hemodialysis; HR, hazard ratio; CI, confidence interval; EP, group treated with short-acting erythropoiesis-stimulating agents; DP, group treated with intermediate-acting erythropoiesis-stimulating agents; MR, group treated with long-acting erythropoiesis-stimulating agents; CCI, Charlson comorbidity index; ESRD, end-stage renal disease; DM, diabetes mellitus; SBP, systolic blood pressure; DBP, diastolic blood pressure; ERI, erythropoietin resistance index; ESA, erythropoiesis-stimulating agent.

Table S2. Cox regression analyses for patient survival in HD patients ≥65 years old.

	Univariate		Multivariate	
	**HR (95% CI)**	***p***	**HR (95% CI)**	***p***
Ref: EP group				
DP group	0.97 (0.93–1.02)	0.244	1.00 (0.95–1.06)	0.871
MR group	0.90 (0.83–0.98)	0.012	0.90 (0.81–0.99)	0.024
Ref: DP group				
MR group	0.92 (0.84–1.01)	0.078	0.89 (0.80–0.99)	0.029
Age (per 1 year increase)	1.06 (1.06–1.07)	<0.001	1.07 (1.06–1.07)	<0.001
Sex (ref: male)	0.82 (0.79–0.85)	<0.001	0.79 (0.75–0.83)	<0.001
CCI score (per 1 score increase)	1.07 (1.06–1.07)	<0.001	1.05 (1.04–1.06)	<0.001
Body mass index (per 1 kg/m^2 increase)	0.96 (0.96–0.97)	<0.001	0.97 (0.96–0.98)	<0.001
Underlying etiology of ESRD (ref: DM)				
Glomerulonephritis	0.61 (0.56–0.66)	<0.001	0.64 (0.57–0.70)	<0.001
Hypertension	0.75 (0.72–0.78)	<0.001	0.70 (0.66–0.74)	<0.001
Others	0.75 (0.69–0.81)	<0.001	0.72 (0.66–0.79)	<0.001
Unknown	0.85 (0.80–0.90)	<0.001	0.76 (0.70–0.83)	<0.001
Hemoglobin (per 1 g/dL increase)	0.89 (0.87–0.91)	<0.001	0.97 (0.94–1.01)	0.125
Serum creatinine (per 1 mg/dL increase)	0.94 (0.93–0.95)	<0.001	0.95 (0.94–0.96)	<0.001
SBP (per 1 mmHg increase)	1.01 (1.00–1.01)	<0.001	1.00 (1.00–1.01)	<0.001
DBP (per 1 mmHg increase)	1.00 (1.00–1.00)	0.457	1.00 (1.00–1.01)	0.011
Serum calcium (per 1 mg/dL increase)	0.99 (0.96–1.01)	0.176	1.06 (1.03–1.09)	<0.001
Serum phosphorus (per 1 mg/dL increase)	0.95 (0.94–0.97)	<0.001	1.02 (1.00–1.04)	0.039
Kt/V_{urea} (per 1 unit increase)	0.82 (0.76–0.88)	<0.001	0.76 (0.69–0.84)	<0.001
Serum albumin (per 1 g/dL increase)	0.51 (0.49–0.54)	<0.001	0.65 (0.61–0.70)	<0.001
HD vintage (per 1 day increase)	1.00 (1.00–1.00)	<0.001	1.00 (1.00–1.00)	<0.001
Ultrafiltration volume (per 1 L increase)	1.05 (1.03–1.07)	<0.001	1.08 (1.05–1.10)	<0.001
ERI (per 1 unit increase)	1.02 (1.02–1.02)	<0.001	1.02 (1.01–1.03)	0.001
ESA dose (per 1 unit/week increase)	1.00 (1.00–1.00)	<0.001	1.00 (1.00–1.00)	0.213

Multivariate analysis was adjusted for group, age, sex, CCI score, body mass index, underlying etiology of ESRD, hemoglobin, serum creatinine, SBP, DBP, serum calcium, serum phosphorus, Kt/V_{urea}, serum albumin, HD vintage, ultrafiltration volume, ERI, and ESA dose, and was performed using enter mode. **Abbreviations**: HD, hemodialysis; HR, hazard ratio; CI, confidence interval; EP, group treated with short-acting erythropoiesis-stimulating agents; DP, group treated with intermediate-acting erythropoiesis-stimulating agents; MR, group treated with long-acting erythropoiesis-stimulating agents; CCI, Charlson comorbidity index; ESRD, end-stage renal disease; DM, diabetes mellitus; SBP, systolic blood pressure; DBP, diastolic blood pressure; ERI, erythropoietin resistance index; ESA, erythropoiesis-stimulating agent.

Table S3. Cox regression analyses for patient survival in male HD patients.

	Univariate		Multivariate	
	**HR (95% CI)**	***p***	**HR (95% CI)**	***p***
Ref: EP group				
DP group	1.05 (1.01–1.10)	0.029	1.03 (0.97–1.08)	0.347
MR group	1.03 (0.94–1.12)	0.545	0.92 (0.83–1.02)	0.109
Ref: DP group				
MR group	0.98 (0.89–1.07)	0.610	0.90 (0.81–0.99)	0.048
Age (per 1 year increase)	1.06 (1.06–1.07)	<0.001	1.06 (1.06–1.06)	<0.001
CCI score (per 1 score increase)	1.13 (1.13–1.14)	<0.001	1.06 (1.05–1.07)	<0.001
Body mass index (per 1 kg/m^2 increase)	0.95 (0.95–0.96)	<0.001	0.98 (0.97–0.99)	<0.001
Underlying etiology of ESRD (ref: DM)				
Glomerulonephritis	0.35 (0.32–0.38)	<0.001	0.54 (0.48–0.60)	<0.001
Hypertension	0.67 (0.64–0.70)	<0.001	0.68 (0.64–0.72)	<0.001
Others	0.59 (0.54–0.63)	<0.001	0.71 (0.65–0.79)	<0.001
Unknown	0.62 (0.58–0.67)	<0.001	0.72 (0.66–0.79)	<0.001
Hemoglobin (per 1 g/dL increase)	0.83 (0.81–0.85)	<0.001	0.94 (0.91–0.98)	<0.001
Serum creatinine (per 1 mg/dL increase)	0.86 (0.85–0.86)	<0.001	0.95 (0.94–0.96)	<0.001
SBP (per 1 mmHg increase)	1.00 (1.00–1.00)	<0.001	1.00 (1.00–1.01)	<0.001
DBP (per 1 mmHg increase)	0.98 (0.98–0.98)	<0.001	1.00 (1.00–1.01)	0.067
Serum calcium (per 1 mg/dL increase)	0.95 (0.93–0.97)	<0.001	1.08 (1.06–1.12)	<0.001
Serum phosphorus (per 1 mg/dL increase)	0.84 (0.83–0.86)	<0.001	1.06 (1.04–1.08)	<0.001
Kt/V_{urea} (per 1 unit increase)	1.38 (1.26–1.51)	<0.001	0.79 (0.71–0.89)	<0.001
Serum albumin (per 1 g/dL increase)	0.35 (0.34–0.37)	<0.001	0.63 (0.59–0.67)	<0.001
HD vintage (per 1 day increase)	1.00 (1.00–1.00)	0.014	1.00 (1.00–1.00)	<0.001
Ultrafiltration volume (per 1 L increase)	0.90 (0.89–0.92)	<0.001	1.06 (1.04–1.09)	<0.001
ERI (per 1 unit increase)	1.03 (1.03–1.03)	<0.001	1.04 (1.03–1.05)	<0.001
ESA dose (per 1 unit/week increase)	1.00 (1.00–1.00)	<0.001	1.00 (1.00–1.00)	<0.001

Multivariate analysis was adjusted for group, age, CCI score, body mass index, underlying etiology of ESRD, hemoglobin, serum creatinine, SBP, DBP, serum calcium, serum phosphorus, Kt/V_{urea}, serum albumin, HD vintage; ultrafiltration volume, ERI, and ESA dose, and was performed using enter mode. **Abbreviations**: HD, hemodialysis; HR, hazard ratio; CI, confidence interval; EP, group treated with short-acting erythropoiesis-stimulating agents; DP, group treated with intermediate-acting erythropoiesis-stimulating agents; MR, group treated with long-acting erythropoiesis-stimulating agents; CCI, Charlson comorbidity index; ESRD, end-stage renal disease; DM, diabetes mellitus; SBP, systolic blood pressure; DBP, diastolic blood pressure; ERI, erythropoietin resistance index; ESA, erythropoiesis-stimulating agent.

Table S4. Cox regression analyses for patient survival in female HD patients.

	Univariate		Multivariate	
	**HR (95% CI)**	***p***	**HR (95% CI)**	***p***
Ref: EP group				
DP group	0.97 (0.91–1.02)	0.229	0.98 (0.91–1.05)	0.531
MR group	0.90 (0.80–1.00)	0.054	0.85 (0.75–0.97)	0.014
Ref: DP group				
MR group	0.93 (0.82–1.05)	0.213	0.87 (0.76–0.99)	0.046
Age (per 1 year increase)	1.07 (1.07–1.07)	<0.001	1.06 (1.06–1.07)	<0.001
CCI score (per 1 score increase)	1.14 (1.13–1.14)	<0.001	1.07 (1.06–1.08)	<0.001
Body mass index (per 1 kg/m^2 increase)	0.99 (0.98–0.99)	0.009	0.97 (0.96–0.99)	<0.001
Underlying etiology of ESRD (ref: DM)				
Glomerulonephritis	0.34 (0.31–0.38)	<0.001	0.51 (0.45–0.58)	<0.001
Hypertension	0.66 (0.62–0.70)	<0.001	0.65 (0.60–0.70)	<0.001
Others	0.48 (0.43–0.52)	<0.001	0.65 (0.57–0.73)	<0.001
Unknown	0.59 (0.55–0.64)	<0.001	0.67 (0.60–0.74)	<0.001
Hemoglobin (per 1 g/dL increase)	0.87 (0.84–0.89)	<0.001	0.95 (0.90–0.99)	0.021
Serum creatinine (per 1 mg/dL increase)	0.82 (0.81–0.83)	<0.001	0.92 (0.91–0.93)	<0.001
SBP (per 1 mmHg increase)	1.01 (1.01–1.01)	<0.001	1.01 (1.00–1.01)	<0.001
DBP (per 1 mmHg increase)	0.99 (0.98–0.99)	<0.001	1.00 (0.99–1.01)	0.067
Serum calcium (per 1 mg/dL increase)	0.93 (0.91–0.96)	<0.001	1.05 (1.01–1.08)	0.009
Serum phosphorus (per 1 mg/dL increase)	0.84 (0.82–0.85)	<0.001	1.02 (0.99–1.04)	0.180
Kt/V_{urea} (per 1 unit increase)	0.81 (0.74–0.89)	<0.001	0.69 (0.62–0.78)	<0.001
Serum albumin (per 1 g/dL increase)	0.38 (0.35–0.40)	<0.001	0.64 (0.59–0.70)	<0.001
HD vintage (per 1 day increase)	1.00 (1.00–1.00)	0.012	1.00 (1.00–1.00)	<0.001
Ultrafiltration volume (per 1 L increase)	0.91 (0.89–0.94)	<0.001	1.07 (1.04–1.10)	<0.001
ERI (per 1 unit increase)	1.01 (1.01–1.02)	<0.001	1.02 (1.01–1.04)	<0.001
ESA dose (per 1 unit/week increase)	1.00 (1.00–1.00)	<0.001	1.00 (1.00–1.00)	0.013

Multivariate analysis was adjusted for group, age, CCI score, body mass index, underlying etiology of ESRD; hemoglobin, serum creatinine, SBP, DBP, serum calcium, serum phosphorus, Kt/V_{urea}, serum albumin, HD vintage, ultrafiltration volume, ERI, and ESA dose, and was performed using enter mode. **Abbreviations**: HD, hemodialysis; HR, hazard ratio; CI, confidence interval; EP, group treated with short-acting erythropoiesis-stimulating agents; DP, group treated with intermediate-acting erythropoiesis-stimulating agents; MR, group treated with long-acting erythropoiesis-stimulating agents; CCI, Charlson comorbidity index; ESRD, end-stage renal disease; DM, diabetes mellitus; SBP, systolic blood pressure; DBP, diastolic blood pressure; ERI, erythropoietin resistance index; ESA, erythropoiesis-stimulating agent.

Table S5. Cox regression analyses for patient survival according to ESA dose tertile in the EP group.

	Univariate		Multivariate	
	**HR (95% CI)**	***p***	**HR (95% CI)**	***p***
Ref: Low tertile				
Middle tertile	1.04 (1.00–1.08)	0.047	1.00 (0.95–1.06)	0.941
High tertile	1.20 (1.16–1.25)	<0.001	1.03 (0.95–1.12)	0.458
Ref: Middle tertile				
High tertile	1.15 (1.11–1.20)	<0.001	1.03 (0.97–1.10)	0.347
Age (per 1 year increase)	1.07 (1.06–1.07)	<0.001	1.06 (1.06–1.06)	<0.001
Sex (ref: male)	0.87 (0.84–0.90)	<0.001	0.77 (0.73–0.81)	<0.001
CCI score (per 1 score increase)	1.14 (1.13–1.14)	<0.001	1.07 (1.06–1.07)	<0.001
Body mass index (per 1 kg/m^2 increase)	0.97 (0.97–0.98)	<0.001	0.98 (0.97–0.99)	<0.001
Underlying etiology of ESRD (ref: DM)				
Glomerulonephritis	0.34 (0.32–0.37)	<0.001	0.53 (0.48–0.58)	<0.001
Hypertension	0.65 (0.62–0.68)	<0.001	0.65 (0.62–0.69)	<0.001
Others	0.52 (0.49–0.56)	<0.001	0.70 (0.64–0.76)	<0.001
Unknown	0.59 (0.56–0.63)	<0.001	0.68 (0.63–0.73)	<0.001
Hemoglobin (per 1 g/dL increase)	0.85 (0.83–0.87)	<0.001	0.94 (0.91–0.97)	<0.001
Serum creatinine (per 1 mg/dL increase)	0.86 (0.86–0.87)	<0.001	0.95 (0.94–0.96)	<0.001
SBP (per 1 mmHg increase)	1.01 (1.01–1.01)	<0.001	1.00 (1.00–1.01)	<0.001
DBP (per 1 mmHg increase)	0.99 (0.98–0.99)	<0.001	1.00 (1.00–1.01)	0.008
Serum calcium (per 1 mg/dL increase)	0.94 (0.92–0.96)	<0.001	1.06 (1.03–1.08)	<0.001
Serum phosphorus (per 1 mg/dL increase)	0.84 (0.83–0.85)	<0.001	1.04 (1.02–1.05)	<0.001
Kt/V_{urea} (per 1 unit increase)	0.89 (0.83–0.95)	<0.001	0.71 (0.64–0.78)	<0.001
Serum albumin (per 1 g/dL increase)	0.37 (0.35–0.39)	<0.001	0.65 (0.61–0.69)	<0.001
HD vintage (per 1 day increase)	1.00 (1.00–1.00)	0.035	1.00 (1.00–1.00)	<0.001
Ultrafiltration volume (per 1 L increase)	0.90 (0.89–0.92)	<0.001	1.06 (1.04–1.09)	<0.001
ERI (per 1 unit increase)	1.02 (1.01–1.02)	<0.001	1.03 (1.02–1.04)	<0.001
ESA dose (per 1 unit/week increase)	1.00 (1.00–1.00)	<0.001	1.00 (1.00–1.00)	<0.001

Multivariate analysis was adjusted for tertile, age, sex, CCI score, body mass index, underlying etiology of ESRD, hemoglobin, serum creatinine, SBP, DBP, serum calcium, serum phosphorus, Kt/V_{urea}, serum albumin, HD vintage, ultrafiltration volume, ERI, and ESA dose, and was performed using enter mode. **Abbreviations**: HD, hemodialysis; EP, group treated with short-acting erythropoiesis-stimulating agents; HR, hazard ratio; CI, confidence interval; CCI, Charlson comorbidity index; ESRD, end-stage renal disease; DM, diabetes mellitus; SBP, systolic blood pressure; DBP, diastolic blood pressure; ERI, erythropoietin resistance index; ESA, erythropoiesis-stimulating agent.

Table S6. Cox regression analyses for patient survival according to ESA dose tertile in the DP group.

	Univariate		Multivariate	
	**HR (95% CI)**	***p***	**HR (95% CI)**	***p***
Ref: Low tertile				
Middle tertile	1.13 (1.04–1.22)	0.003	0.97 (0.88–1.07)	0.545
High tertile	1.34 (1.24–1.45)	<0.001	0.94 (0.82–1.08)	0.405
Ref: Middle tertile				
High tertile	1.19 (1.11–1.28)	<0.001	0.97 (0.87–1.09)	0.629
Age (per 1 year increase)	1.07 (1.06–1.07)	<0.001	1.06 (1.06–1.06)	<0.001
Sex (ref: male)	0.80 (0.75–0.86)	<0.001	0.70 (0.64–0.77)	<0.001
CCI score (per 1 score increase)	1.14 (1.12–1.15)	<0.001	1.06 (1.04–1.07)	<0.001
Body mass index (per 1 kg/m^2 increase)	0.97 (0.97–0.98)	<0.001	0.97 (0.96–0.99)	<0.001
Underlying etiology of ESRD (ref: DM)				
Glomerulonephritis	0.35 (0.31–0.41)	<0.001	0.51 (0.43–0.61)	<0.001
Hypertension	0.71 (0.66–0.77)	<0.001	0.71 (0.64–0.78)	<0.001
Others	0.54 (0.48–0.61)	<0.001	0.68 (0.58–0.79)	<0.001
Unknown	0.62 (0.55–0.70)	<0.001	0.73 (0.63–0.86)	<0.001
Hemoglobin (per 1 g/dL increase)	0.85 (0.81–0.88)	<0.001	0.94 (0.89–1.00)	0.057
Serum creatinine (per 1 mg/dL increase)	0.86 (0.85–0.87)	<0.001	0.93 (0.92–0.95)	<0.001
SBP (per 1 mmHg increase)	1.01 (1.00–1.01)	<0.001	1.00 (1.00–1.01)	0.001
DBP (per 1 mmHg increase)	0.98 (0.97–0.98)	<0.001	1.00 (0.99–1.00)	0.934
Serum calcium (per 1 mg/dL increase)	0.93 (0.90–0.97)	<0.001	1.13 (1.08–1.19)	<0.001
Serum phosphorus (per 1 mg/dL increase)	0.86 (0.84–0.88)	<0.001	1.07 (1.03–1.10)	<0.001
Kt/V_{urea} (per 1 unit increase)	0.99 (0.88–1.11)	0.887	0.87 (0.74–1.02)	0.087
Serum albumin (per 1 g/dL increase)	0.35 (0.32–0.38)	<0.001	0.59 (0.53–0.67)	<0.001
HD vintage (per 1 day increase)	1.00 (1.00–1.00)	0.018	1.00 (1.00–1.00)	<0.001
Ultrafiltration volume (per 1 L increase)	0.95 (0.92–0.98)	0.002	1.06 (1.01–1.10)	0.018
ERI (per 1 unit increase)	1.02 (1.02–1.03)	<0.001	1.01 (0.99–1.03)	0.351
ESA dose (per 1 unit/week increase)	1.00 (1.00–1.00)	<0.001	1.00 (1.00–1.00)	0.329

Multivariate analysis was adjusted for tertile, age, sex, CCI score, body mass index, underlying etiology of ESRD, hemoglobin, serum creatinine, SBP, DBP, serum calcium, serum phosphorus, Kt/V_{urea}, serum albumin, HD vintage, ultrafiltration volume, ERI, and ESA dose, and was performed using enter mode. **Abbreviations**: DP, group treated with intermediate-acting erythropoiesis-stimulating agents; HR, hazard ratio; CI, confidence interval; CCI, Charlson comorbidity index; ESRD, end-stage renal disease; DM, diabetes mellitus; SBP, systolic blood pressure; DBP, diastolic blood pressure; ERI, erythropoietin resistance index; ESA, erythropoiesis-stimulating agent.

Table S7. Cox regression analyses for patient survival according to ESA dose tertile in the MR group.

	Univariate		Multivariate	
	**HR (95% CI)**	***p***	**HR (95% CI)**	***p***
Ref: Low tertile				
Middle tertile	0.94 (0.80–1.11)	0.475	1.05 (0.85–1.30)	0.670
High tertile	1.26 (1.07–1.48)	0.004	1.34 (0.99–1.81)	0.056
Ref: Middle tertile				
High tertile	1.34 (1.14–1.58)	<0.001	1.28 (1.01–1.61)	0.039
Age (per 1 year increase)	1.06 (1.06–1.07)	<0.001	1.06 (1.05–1.07)	<0.001
Sex (ref: male)	0.76 (0.66–0.87)	<0.001	0.68 (0.55–0.83)	<0.001
CCI score (per 1 score increase)	1.13 (1.11–1.16)	<0.001	1.07 (1.04–1.10)	<0.001
Body mass index (per 1 kg/m^2 increase)	0.98 (0.96–1.00)	0.095	1.02 (0.98–1.05)	0.340
Underlying etiology of ESRD (ref: DM)				
Glomerulonephritis	0.33 (0.24–0.45)	<0.001	0.55 (0.37–0.82)	0.002
Hypertension	0.71 (0.60–0.83)	<0.001	0.70 (0.58–0.86)	<0.001
Others	0.56 (0.42–0.75)	<0.001	0.56 (0.39–0.81)	0.002
Unknown	0.71 (0.56–0.91)	0.006	0.90 (0.67–1.20)	0.465
Hemoglobin (per 1 g/dL increase)	0.87 (0.80–0.95)	0.001	1.00 (0.88–1.14)	0.995
Serum creatinine (per 1 mg/dL increase)	0.87 (0.85–0.90)	<0.001	0.94 (0.90–0.98)	0.003
SBP (per 1 mmHg increase)	1.01 (1.00–1.01)	0.040	1.00 (0.99–1.01)	0.198
DBP (per 1 mmHg increase)	0.98 (0.97–0.99)	<0.001	1.00 (0.99–1.01)	0.464
Serum calcium (per 1 mg/dL increase)	0.85 (0.78–0.92)	<0.001	1.11 (1.00–1.24)	0.046
Serum phosphorus (per 1 mg/dL increase)	0.86 (0.82–0.91)	<0.001	1.05 (0.98–1.12)	0.137
Kt/V_{urea} (per 1 unit increase)	0.86 (0.67–1.12)	0.264	0.64 (0.45–0.91)	0.014
Serum albumin (per 1 g/dL increase)	0.32 (0.27–0.39)	<0.001	0.61 (0.48–0.77)	<0.001
HD vintage (per 1 day increase)	1.00 (1.00–1.00)	0.951	1.00 (1.00–1.00)	<0.001
Ultrafiltration volume (per 1 L increase)	0.93 (0.87–0.99)	0.042	1.11 (1.01–1.22)	0.030
ERI (per 1 unit increase)	1.04 (1.03–1.06)	<0.001	1.16 (1.07–1.26)	<0.001
ESA dose (per 1 unit/week increase)	1.00 (1.00–1.00)	<0.001	0.99 (0.99–0.99)	<0.001

Multivariate analysis was adjusted for tertile, age, sex, CCI score, body mass index, underlying etiology of ESRD, hemoglobin, serum creatinine, SBP, DBP, serum calcium, serum phosphorus, Kt/V_{urea}, serum albumin, HD vintage, ultrafiltration volume, ERI, and ESA dose, and was performed using enter mode. **Abbreviations**: MR, group treated with long-acting erythropoiesis-stimulating agents; HR, hazard ratio; CI, confidence interval; CCI, Charlson comorbidity index; ESRD, end-stage renal disease; DM, diabetes mellitus; SBP, systolic blood pressure; DBP, diastolic blood pressure; ERI, erythropoietin resistance index; ESA, erythropoiesis-stimulating agent.

Table S8. Linear regression analyses to identify factors associated with ERI.

	Univariate		Multivariate	
	**St-β ± SE**	***p***	**St-β ± SE**	***p***
Ref: EP group				
DP group	–0.794 ± 0.076	<0.001	0.068 ± 0.025	0.006
MR group	–4.421 ± 0.148	<0.001	0.031 ± 0.046	0.506
Ref: DP group				
MR group	–3.627 ± 0.159	<0.001	–0.038 ± 0.049	0.446
Age (per 1 year increase)	0.039 ± 0.002	<0.001	0.005 ± 0.001	<0.001
Sex (ref: male)	2.365 ± 0.061	<0.001	1.196 ± 0.025	<0.001
CCI score (per 1 score increase)	0.106 ± 0.011	<0.001	0.018 ± 0.004	<0.001
Body mass index (per 1 kg/m^2 increase)	–0.489 ± 0.009	<0.001	–0.399 ± 0.003	<0.001
Underlying etiology of ESRD (ref: DM)				
Glomerulonephritis	0.677 ± 0.106	<0.001	0.169 ± 0.037	<0.001
Hypertension	0.680 ± 0.075	<0.001	0.204 ± 0.027	<0.001
Others	0.788 ± 0.116	<0.001	0.238 ± 0.040	<0.001
Unknown	0.879 ± 0.105	<0.001	0.265 ± 0.037	<0.001
Hemoglobin (per 1 g/dL increase)	–4.335 ± 0.039	<0.001	–1.018 ± 0.015	<0.001
Serum creatinine (per 1 mg/dL increase)	–0.231 ± 0.011	<0.001	–0.065 ± 0.005	<0.001
SBP (per 1 mmHg increase)	0.017 ± 0.002	<0.001	0.002 ± 0.001	0.016
DBP (per 1 mmHg increase)	–0.007 ± 0.004	0.079	–0.001 ± 0.001	0.364
Serum calcium (per 1 mg/dL increase)	–0.217 ± 0.038	<0.001	–0.043 ± 0.012	<0.001
Serum phosphorus (per 1 mg/dL increase)	–0.192 ± 0.023	<0.001	–0.020 ± 0.008	0.015
Kt/V_{urea} (per 1 unit increase)	3.448 ± 0.122	<0.001	1.076 ± 0.046	<0.001
Serum albumin (per 1 g/dL increase)	–2.612 ± 0.088	<0.001	–0.179 ± 0.031	<0.001
HD vintage (per 1 day increase)	0.000 ± 0.000	<0.001	0.000 ± 0.000	<0.001
Ultrafiltration volume (per 1 L increase)	–0.037 ± 0.032	0.253	–0.097 ± 0.011	<0.001
ESA dose (per 1 unit/week increase)	0.002 ± 0.000	<0.001	0.002 ± 0.000	<0.001

Multivariate analysis was adjusted for group, age, sex, CCI score, body mass index, underlying etiology of ESRD, hemoglobin, serum creatinine, SBP, DBP, serum calcium, serum phosphorus, Kt/V_{urea}, serum albumin, HD vintage, ultrafiltration volume, and ESA dose, and was performed using enter mode. **Abbreviations**: St-β, standardized β; SE, standard error; HD, hemodialysis; HR, hazard ratio; CI, confidence interval; EP, group treated with short-acting erythropoiesis-stimulating agents; DP, group treated with intermediate-acting erythropoiesis-stimulating agents; MR, group treated with long-acting erythropoiesis-stimulating agents; CCI, Charlson comorbidity index; ESRD, end-stage renal disease; DM, diabetes mellitus; SBP, systolic blood pressure; DBP, diastolic blood pressure; ERI, erythropoietin resistance index; ESA, erythropoiesis-stimulating agent.
